# Clues to Neuro-Degeneration in Niemann-Pick Type C Disease from Global Gene Expression Profiling

**DOI:** 10.1371/journal.pone.0000019

**Published:** 2006-12-20

**Authors:** Jonathan V. Reddy, Ian G. Ganley, Suzanne R. Pfeffer

**Affiliations:** Department of Biochemistry, Stanford University School of Medicine Stanford, California, United States of America; University of the Western Cape, South Africa

## Abstract

**Background:**

Niemann-Pick Type C (NPC) disease is a neurodegenerative disease that is characterized by the accumulation of cholesterol and glycosphingolipids in the late endocytic pathway. The majority of NPC cases are due to mutations in the NPC1 gene. The precise function of this gene is not yet known.

**Methodology/Principal Findings:**

Using cDNA microarrays, we analyzed the genome-wide expression patterns of human fibroblasts homozygous for the I1061T NPC1 mutation that is characterized by a severe defect in the intracellular processing of low density lipoprotein-derived cholesterol. A distinct gene expression profile was identified in NPC fibroblasts from different individuals when compared with fibroblasts isolated from normal subjects. As expected, NPC1 mutant cells displayed an inappropriate homeostatic response to accumulated intracellular cholesterol. In addition, a number of striking parallels were observed between NPC disease and Alzheimer's disease.

**Conclusions/Significance:**

Many genes involved in the trafficking and processing of amyloid precursor protein and the microtubule binding protein, tau, were more highly expressed. Numerous genes important for membrane traffic and the cellular regulation of calcium, metals and other ions were upregulated. Finally, NPC fibroblasts exhibited a gene expression profile indicative of oxidative stress. These changes are likely contributors to the pathophysiology of Niemann-Pick Type C disease.

## Introduction

Niemann Pick Type C disease (NPC) is a fatal, autosomal recessive neurodegenerative disease due to mutations in the NPC1 or NPC2 genes [1], [2]. NPC is associated with an inability to process cellular cholesterol, which accumulates together with glycosphingolipids within lysosomes of affected individuals. The disease is often diagnosed in early childhood, with patients typically displaying cerebellar ataxia, difficulty speaking and swallowing, and progressive dementia. Mutations in the NPC1 gene account for approximately 95% of NPC cases.

NPC1 is a 1278 amino acid protein with 13 transmembrane domains that is important for normal cholesterol homeostasis [3]. NPC1 transmembrane domains 3–7 comprise a so-called sterol sensing domain that is related to hydroxymethlyglutaryl CoA reductase [4], sterol regulatory element-binding protein cleavage activating protein (SCAP; [5], PATCHED [6], [7]) and NPC1-like protein, NPC1L1 [8]. The NPC1 protein was recently shown to bind cholesterol [9]. Mutations in the NPC1 sterol sensing domain can cause disease, as can mutations throughout the protein, the bulk of which resides within the membrane bilayer or within the lumen of late endosomes [1].

To begin to understand how loss of NPC1 function leads to neurodegeneration, we have examined the global pattern of gene expression in primary fibroblast cells from NPC patients. To avoid possible phenotypic variation between different mutant alleles, we designed our analysis using cells from different individuals who each carried the identical, homozygous mutations within their NPC1 loci. The most common NPC-1 mutation described is I1061T, which accounts for 20% of the alleles in the United Kingdom and France and about 15% in the United States [10], [11] and leads to a severe disruption of cholesterol processing. Homozygous carriers of the I1061T mutation present with a relatively mild neurological form of the disease with onset in juvenile years; importantly, their clinical presentation is homogeneous. In our experimental design, we hoped that gene expression differences between any two individuals would be averaged out, highlighting changes due to this specific mutation at both NPC1 alleles. As shown here, NPC1 fibroblasts showed highly significant differences when compared with normal fibroblast controls. The specific gene expression changes observed provide important clues to the pathophysiology of NPC disease.

## Results and Discussion

**Figure 1:**
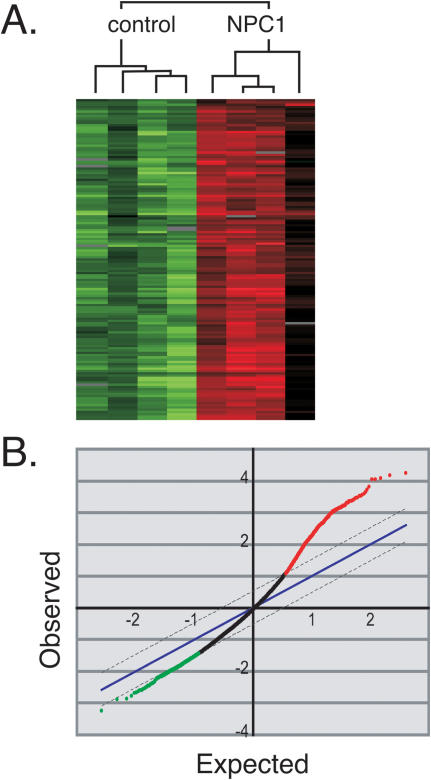
Gene expression is significantly altered in NPC1 fibroblasts. (A) Supervised hierarchical clustering of the top 200 genes after SAM analysis. The dendrogram at the top shows clustering of NPC and control fibroblasts into distinct groups. Genes with higher expression are shown in red; genes with lower expression are shown in green. Gray bars indicate missing data. Cell lines clustered in the following order (left to right): control lines AG10803, GM05659, GM09503, GM03726; NPC-1 lines GM017909, 59413, 83.16, and GM18453. (B) Significance Analysis of Microarrays [12] was used to identify genes that varied in expression between NPC fibroblasts and control fibroblasts from healthy patients. The plot of the data from 43,000 elements reveals approximately 3600 significant genes that have increased expression in NPC fibroblasts (shown in red).

Primary skin fibroblasts isolated from four patients homozygous for the I1061T mutation and four control individuals were cultured under identical conditions. RNA was isolated from all samples and hybridized to cDNA microarrays consisting of approximately 43,000 elements representing approximately 36,000 genes. Analysis of the data using a supervised hierarchical clustering method identified a gene expression pattern that was unique and consistent among NPC fibroblasts (Figure 1A). To identify statistically significant gene expression profile changes that are likely to result from the I1061T mutation in the NPC1 gene we used SAM (Significance Analysis of Microarrays; [12]).

SAM identifies genes with statistically significant changes in expression by assimilating a set of gene-specific *t* tests. Each gene is assigned a score on the basis of its change in gene expression relative to the standard deviation of repeated measurements for that gene. Genes with scores greater than a threshold are deemed potentially significant. The percentage of such genes identified by chance is defined as the false discovery rate. Figure 1B shows the distribution of the ∼43,000 elements analyzed in this study. Many more genes were found to be significantly up-regulated (3578, red points) than down-regulated (699, green points) when comparing normal and NPC1 cells (Fig. 1B). SAM identified approximately 3600 significant genes in our data set with an estimated false discovery rate of 5%; ∼1300 genes showed ≥1.5 fold up-regulation compared with normal cells. The large number of upregulated genes suggests that NPC cells are compensating to survive under challenging circumstances. An Excel list of all significant genes showing a <2% false discovery rate is included for the reader as a supplemental file (Dataset S1).

It is important to note that the absolute fold change in the level of a specific transcript need not be high to be highly significant, and to have significant consequences for cell physiology [12]. For many of the genes shown, the potential false discovery rate was less than 1%, and as described below, several of the changes have been verified at the protein level.

Initial analysis of the upregulated genes confirmed the validity of our experimental protocol, as genes expected to be upregulated in cells lacking NPC1 function were upregulated: for example, the gene encoding NPC2 protein was upregulated 1.7 fold and the gene encoding NPC1-like protein, NPC1L1 was upregulated 1.3 fold (Table 1). This confirms previous work demonstrating the upregulation of NPC2 at the protein level in NPC1 fibroblasts [13], [14].

**Table 1:**
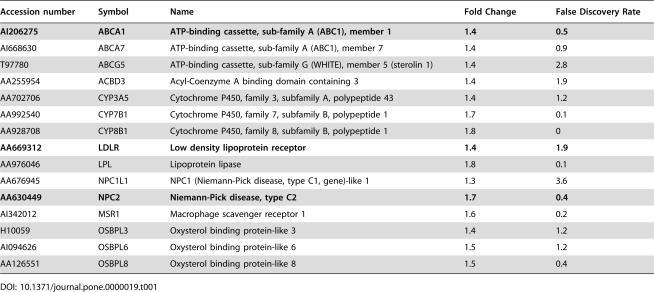
Genes involved in Cholesterol Homeostasis are upregulated in NPC cells.

Accession number	Symbol	Name	Fold Change	False Discovery Rate
**AI206275**	**ABCA1**	**ATP-binding cassette, sub-family A (ABC1), member 1**	**1.4**	**0.5**
AI668630	ABCA7	ATP-binding cassette, sub-family A (ABC1), member 7	1.4	0.9
T97780	ABCG5	ATP-binding cassette, sub-family G (WHITE), member 5 (sterolin 1)	1.4	2.8
AA255954	ACBD3	Acyl-Coenzyme A binding domain containing 3	1.4	1.9
AA702706	CYP3A5	Cytochrome P450, family 3, subfamily A, polypeptide 43	1.4	1.2
AA992540	CYP7B1	Cytochrome P450, family 7, subfamily B, polypeptide 1	1.7	0.1
AA928708	CYP8B1	Cytochrome P450, family 8, subfamily B, polypeptide 1	1.8	0
**AA669312**	**LDLR**	**Low density lipoprotein receptor**	**1.4**	**1.9**
AA976046	LPL	Lipoprotein lipase	1.8	0.1
AA676945	NPC1L1	NPC1 (Niemann-Pick disease, type C1, gene)-like 1	1.3	3.6
**AA630449**	**NPC2**	**Niemann-Pick disease, type C2**	**1.7**	**0.4**
AI342012	MSR1	Macrophage scavenger receptor 1	1.6	0.2
H10059	OSBPL3	Oxysterol binding protein-like 3	1.4	1.2
AI094626	OSBPL6	Oxysterol binding protein-like 6	1.5	1.2
AA126551	OSBPL8	Oxysterol binding protein-like 8	1.5	0.4

### Cholesterol Homeostasis

In normal cells, cholesterol levels are tightly regulated by a number of mechanisms that include cholesterol uptake, storage and efflux [15]. At the transcriptional level, sterol regulatory element-binding proteins regulate the expression of a large number of genes involved in the synthesis and uptake of cholesterol, fatty acids, triglycerides and phospholipids, as well as NADPH that is needed for lipid and cholesterol synthesis [16]. In the presence of adequate cholesterol in the endoplasmic reticulum (ER), these transcription factors fail to translocate to the nucleus, and genes involved in cholesterol synthesis and uptake are no longer activated.

NPC cells are well documented in terms of their inability to respond to the accumulation of unesterified cholesterol within late endocytic compartments. In these cells, LDL receptor expression is not down-regulated and consequently, LDL uptake continues to occur despite the increased cellular content of free cholesterol [17]. Microarray analysis confirmed this dysregulation: elevated expression (1.5 fold) of the LDL receptor gene was readily detected in NPC fibroblasts homozygous for the I1061T mutation when compared with normal fibroblasts (Table 1).

Cellular cholesterol is also regulated by Liver X Receptors (LXRs), which are activated by cholesterol-derived oxysterols. LXRs activate target genes that participate in the efflux of cellular cholesterol, including the transporters, ABCA1 and ABCG5/ABCG8. NPC cells are defective in oxysterol generation and subsequent LXR activation [18]. Previous, direct measurements of ABCA1 mRNA indicated significantly less upregulation of ABCA1 gene expression in NPC1^−/−^ cells upon cholesterol addition than observed in wild type cells [19]. ABCA1 and ABCG5 expression levels were altered in NPC cells in accordance with earlier reports (Table 1). In addition, we observed increased expression of ABCA7, a non-LXR regulated transporter that has the ability to support apolipoprotein-mediated release of cellular cholesterol and phospholipid (Table 1, ref. [20]).

Increased expression of several proteins from the oxysterol binding protein family (OSBPL3, OSBPL6 and OSBPL8) was also detected (Table 1). One function ascribed to this protein family is non-vesicular cholesterol transfer between the ER and the plasma membrane [21]. Both OSBPL3 and OSBPL6 contain targeting sequences for the ER and plasma membrane. Interestingly, overexpression of the related OSBP2 protein leads to the activation of cholesterol synthesis and LDL receptor upregulation, perhaps by enhancing ER cholesterol efflux [21].

### Links to Neurodegenerative Disease

As in Alzheimer's disease, defects in the processing of the amyloid precursor protein (APP) are well documented for NPC cells [22]–[25]. APP can be processed via two different pathways. In the non-amyloidogenic pathway, APP is cleaved by alpha-secretase to generate a C-terminal fragment (CTF) followed by cleavage with the gamma-secretase complex, resulting in the generation of two non-amyloidogenic peptides. In the amyloidogenic pathway, APP is cleaved by beta-secretase prior to cleavage with gamma-secretase, generating the Abeta fragments that are associated with amyloid plaque formation. Burns et al. [23] have shown that mouse NPC cells show enhanced gamma secretase activity, leading to increased Abeta, Abeta42 and beta-CTF production. They also noted an alteration in the localization of presenilin 1 to some Rab5-positive, putative early endosomes in NPC cells [23]. Increased levels of Abeta, Abeta 42 and beta-CTF were also reported by others [22], [24], [25].

**Figure 2:**
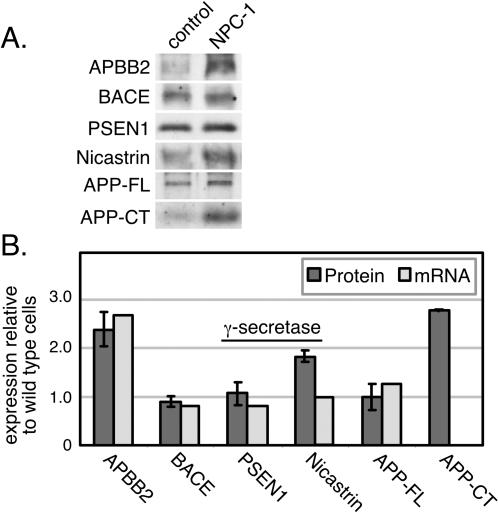
Links to Alzheimer's disease at the mRNA and protein levels in NPC Cells. Protein levels in control and NPC-1 fibroblasts, determined by immunoblot (panel A), are quantified in B (dark shaded bars); mRNA levels from microarray data are shown in light shaded bars for comparison.

Consistent with previous analyses of mouse NPC cells [23], we found little difference in the mRNA and protein levels of full length amyloid precursor protein, beta secretases (BACE), or the level of the gamma secretase component, presenilin, in human NPC1 cells compared with normal fibroblasts (Figure 2). At the protein level, Nicastrin polypeptide was increased, which could contribute to increased gamma secretase activity. Importantly, we confirmed a significant increase in the generation of amyloid C-terminal fragments at the protein level, in human NPC-1 fibroblasts (Figure 2).

Additionally, expression of the amyloid beta precursor binding protein 2 (APBB2 or Fe65-like), was upregulated 2.6 fold with high significance (0.1% false discovery rate), and was among the highest upregulated genes in our entire data set (Table 2). This protein is thought to regulate the intracellular transport of the amyloid precursor protein. As shown in Figure 2, both the mRNA and APBB2 protein were upregulated ∼2.5 fold in NPC1 cells as determined by immunoblotting of cell extracts. APBB2 interacts with the cytoplasmic domain of APP [26], [27]. The association of APBB2 with APP is required for subsequent processing of APP CTF's by gamma secretases, and overexpression of APBB2 leads to the increased generation of the amyloidogenic Abeta42 fragment [27]. Upregulation of APBB2 in NPC fibroblasts may contribute to the increased generation of Abeta42 in NPC cells.

**Table 2:**
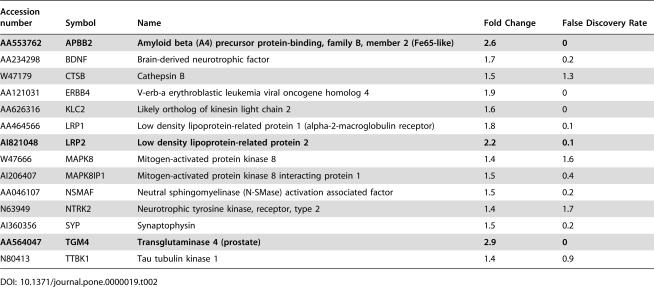
Genes associated with Alzheimer's disease are upregulated in NPC cells.

Accession number	Symbol	Name	Fold Change	False Discovery Rate
**AA553762**	**APBB2**	**Amyloid beta (A4) precursor protein-binding, family B, member 2 (Fe65-like)**	**2.6**	**0**
AA234298	BDNF	Brain-derived neurotrophic factor	1.7	0.2
W47179	CTSB	Cathepsin B	1.5	1.3
AA121031	ERBB4	V-erb-a erythroblastic leukemia viral oncogene homolog 4	1.9	0
AA626316	KLC2	Likely ortholog of kinesin light chain 2	1.6	0
AA464566	LRP1	Low density lipoprotein-related protein 1 (alpha-2-macroglobulin receptor)	1.8	0.1
**AI821048**	**LRP2**	**Low density lipoprotein-related protein 2**	**2.2**	**0.1**
W47666	MAPK8	Mitogen-activated protein kinase 8	1.4	1.6
AI206407	MAPK8IP1	Mitogen-activated protein kinase 8 interacting protein 1	1.5	0.4
AA046107	NSMAF	Neutral sphingomyelinase (N-SMase) activation associated factor	1.5	0.2
N63949	NTRK2	Neurotrophic tyrosine kinase, receptor, type 2	1.4	1.7
AI360356	SYP	Synaptophysin	1.5	0.2
**AA564047**	**TGM4**	**Transglutaminase 4 (prostate)**	**2.9**	**0**
N80413	TTBK1	Tau tubulin kinase 1	1.4	0.9

Recent work has indicated that Abeta42 may directly activate neutral sphingomyelinases [28]. This activation would potentially increase ceramide generation in NPC1 cells and may be linked to oxidative stress. A neutral sphingomyelinase activating protein mRNA (NSMAF) was upregulated in our dataset (Table 2).

Genes encoding LDL receptor-related proteins (LRP1, LRP2 and LRP6) were upregulated 1.8, 2.2 and 2.7 fold, respectively, in NPC fibroblasts (Table 2 and Supp. Table 3). LRP1 interacts with APP and may enhance its internalization, thereby favoring initial intracellular APP cleavage by beta-secretase [29]. APP undergoes axonal transport to nerve terminals in a complex with the signaling protein, MAPK8IP1 in a kinesin-1 dependent manner [30], [31]. A Kinesin-1 component (KLC2) and MAPK8IP1 were all upregulated in NPC cells, and could contribute to the transport of APP in NPC neurons (Table 2). In summary, genes encoding multiple factors that contribute to the generation of amyloid are expressed at higher levels in NPC1 cells.

**Table 3:**
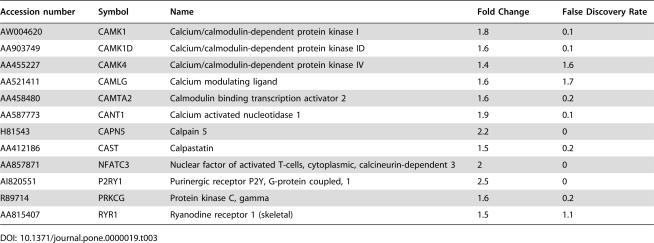
Genes associated with calcium regulation are upregulated in NPC fibroblasts.

Accession number	Symbol	Name	Fold Change	False Discovery Rate
AW004620	CAMK1	Calcium/calmodulin-dependent protein kinase I	1.8	0.1
AA903749	CAMK1D	Calcium/calmodulin-dependent protein kinase ID	1.6	0.1
AA455227	CAMK4	Calcium/calmodulin-dependent protein kinase IV	1.4	1.6
AA521411	CAMLG	Calcium modulating ligand	1.6	1.7
AA458480	CAMTA2	Calmodulin binding transcription activator 2	1.6	0.2
AA587773	CANT1	Calcium activated nucleotidase 1	1.9	0.1
H81543	CAPN5	Calpain 5	2.2	0
AA412186	CAST	Calpastatin	1.5	0.2
AA857871	NFATC3	Nuclear factor of activated T-cells, cytoplasmic, calcineurin-dependent 3	2	0
AI820551	P2RY1	Purinergic receptor P2Y, G-protein coupled, 1	2.5	0
R89714	PRKCG	Protein kinase C, gamma	1.6	0.2
AA815407	RYR1	Ryanodine receptor 1 (skeletal)	1.5	1.1

### Intracellular Calcium Regulation

The expression of a number of calcium-regulated and regulating proteins was increased in NPC fibroblasts (Table 3). These include voltage gated calcium channels such as CACNA1C, calcium dependent protein kinases (CAMK1 and CAMK4), phospholipase C, ryanodine receptor and also calpains (CAPN5). The upregulation of these proteins is consistent with altered cytosolic calcium levels in NPC1 mutant cells. Defects in calcium regulation have been reported for a number of neurodegenerative diseases (see [32], [33] for review). Moreover, Abeta42 has been shown to disrupt intracellular calcium regulation which may play a role in Alzheimer's disease [34]. Abeta increases the activation of L type voltage gated calcium channels [35], [36] and has even been proposed to itself form a cation channel [37].

#### Tau protein

One of the major features of both Alzheimer's and NPC diseases is the accumulation of neurofibrillary tangles in the brain. A major component of such tangles is an abnormally phosphorylated form of the microtubule binding protein, tau [38]. Although the mechanism of tangle formation is unknown, phosphorylation of the tau protein C-terminus is thought to disrupt microtubule binding and trigger subsequent aggregation. NPC fibroblasts displayed enhanced expression of a number of kinases that may participate in tau phosphorylation (TTBK1, MAPK8, PRKCG) (Table 2, III; [39]–[41]). Divalent cations such as calcium have also been implicated in the formation of phosphorylated tau aggregates [42]. As discussed earlier, many alterations in gene expression suggest an alteration in calcium homeostasis in NPC cells (Table 3).

Transglutaminase catalyzes the formation of insoluble tau aggregates in vitro, and increased transglutaminase activity has been observed in Alzheimer's disease [43], [44]. Transglutaminase 4 is significantly upregulated in NPC fibroblasts, and may contribute to tau aggregation in NPC disease [Table 2).

#### Cellular toxicity induced in NPC disease

Oxidative stress is a major factor in a variety of neurodegenerative diseases [45]. NPC cells displayed a number of changes in gene expression that are associated with the generation of reactive oxygen and nitrogen species (Table 4). CHGA (chromogranin A), a component of Alzheimer's disease plaques [46], induces nitric oxide efflux from microglia, a key step in neurodegeneration [47]. Components of the phagocytic NADPH oxidase (CYBB, NCF4) that can generate reactive oxygen species were upregulated [48]. Correspondingly, a number of genes that respond to oxidative stress (MT1G, MT3, GPX3 and GSTM2) were also upregulated in NPC fibroblasts.

**Table 4:**
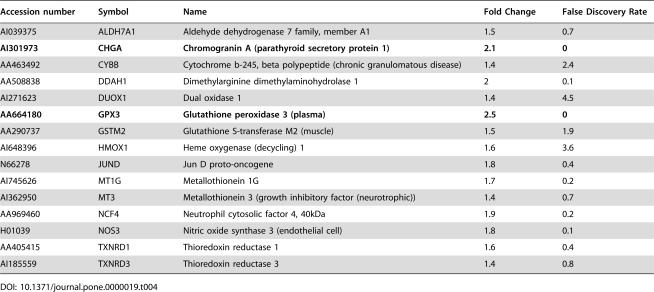
Genes involved in oxidative stress that are upregulated in NPC fibroblasts.

Accession number	Symbol	Name	Fold Change	False Discovery Rate
AI039375	ALDH7A1	Aldehyde dehydrogenase 7 family, member A1	1.5	0.7
**AI301973**	**CHGA**	**Chromogranin A (parathyroid secretory protein 1)**	**2.1**	**0**
AA463492	CYBB	Cytochrome b-245, beta polypeptide (chronic granulomatous disease)	1.4	2.4
AA508838	DDAH1	Dimethylarginine dimethylaminohydrolase 1	2	0.1
AI271623	DUOX1	Dual oxidase 1	1.4	4.5
**AA664180**	**GPX3**	**Glutathione peroxidase 3 (plasma)**	**2.5**	**0**
AA290737	GSTM2	Glutathione S-transferase M2 (muscle)	1.5	1.9
AI648396	HMOX1	Heme oxygenase (decycling) 1	1.6	3.6
N66278	JUND	Jun D proto-oncogene	1.8	0.4
AI745626	MT1G	Metallothionein 1G	1.7	0.2
AI362950	MT3	Metallothionein 3 (growth inhibitory factor (neurotrophic))	1.4	0.7
AA969460	NCF4	Neutrophil cytosolic factor 4, 40kDa	1.9	0.2
H01039	NOS3	Nitric oxide synthase 3 (endothelial cell)	1.8	0.1
AA405415	TXNRD1	Thioredoxin reductase 1	1.6	0.4
AI185559	TXNRD3	Thioredoxin reductase 3	1.4	0.8

Components of the mitochondrial electron transport chain were also upregulated. This may indicate a compensatory response to a disruption in electron transport chain function as seen in other neurodegenerative diseases such as Parkinson's. Reduced electron transport chain activity has been associated with reactive oxygen species production [49].

Altered levels of the metal ions copper, zinc and iron have been observed in Alzheimer's disease. The disruption in steady state levels of iron and copper may play a role in oxidative damage [50], [51]. NPC fibroblasts demonstrated enhanced expression of a wide range of transporters and solute carriers, suggesting that the cells are electro-physiologically challenged (Table S1). Genes involved in iron homeostasis and copper and zinc transport were also upregulated, suggestive of an alteration in the cellular homeostasis of these cations (Table S2).

### Pleiotropic Changes in Membrane Trafficking Factors

The increased cholesterol in NPC late endosomes and lysosomes interferes with transport of proteins between these compartments (cf. [52]). Consequently, proteins involved in membrane trafficking and in particular, lysosome biogenesis, may be upregulated to compensate for this defect. The lysosomal hydrolase, cathepsin B, was upregulated in NPC cells. Both cathepsin B and cathepsin D can function as beta-secretase enzymes, and are partially mislocalized to early endosomes in Alzheimer's disease [53], [54]. We have shown elsewhere that in NPC cells, the cation-independent mannose 6-phosphate receptor (CI-MPR) is upregulated 1.6 fold at the protein level, along with two proteins involved in its recycling to the Golgi complex: Rab9 GTPase and the Rab9 effector, p40 [52]. p40 mRNA showed a 1.3 fold upregulation in our dataset (Table S3); CI-MPR and Rab9 were not present among the final array data elements. The cation-dependent (CD)-MPR was upregulated 1.5 fold in our microarray analyses (Table S3). This may be in part to compensate for this protein's rapid degradation in NPC fibroblasts [52].

In addition to the MPR trafficking components, many key factors required for membrane traffic were upregulated (Table S3). This includes as many as 8 Rab GTPases and numerous Rab interacting proteins and GTPase activating proteins (TBC-domain containing proteins), and several coat complexes involved in transport vesicle assembly. Rab proteins are general regulators of membrane traffic and have been of particular interest in NPC-disease, as overexpression of several Rabs appears to reverse the cholesterol accumulation phenotype in cell culture [reviewed in 52]. The BLOC1S1 protein complex that is key in the biogenesis of lysosome related organelles was upregulated 2.4 fold (Table S3), and an AP3-subunit key for synaptic vesicle recycling was also upregulated 1.3 fold. Loss of AP3 leads to hippocampal granule deficiency in mice deficient in this component [55]. A number of so-called SNARE proteins and general membrane traffic components including SEC22 and SEC24 were also upregulated. These latter two proteins participate in protein transport from the endoplasmic reticulum to the Golgi complex.

Multiple kinesin, myosin and dynein motors were upregulated, perhaps to compensate for the inhibition of vesicle motility characteristic of cells lacking NPC1 function [56], [57]. Additional cytoskeletal regulatory proteins including annexin A13 and A6, ankyrin2, several Rho GTPase activating proteins and actin related protein 3 were all upregulated. Many of these changes likely reflect compensatory responses to cholesterol and sphingolipid accumulation-triggered defects.

## Conclusions

Gene expression analysis of fibroblasts from patients with Niemann-Pick type C have revealed many intriguing similarities with classic neurodegenerative diseases. Further study of newly identified candidate genes are likely to reveal important clues to the pathophysiology of this genetic disorder.

## Materials and Methods

### Cells

NPC1^I1061T^ homozygous fibroblasts GM18453, GM17909 and the heterozygous GM03123 fibroblast cell line containing the I1061T mutation were obtained from Coriell cell repositories (Camden, NJ.) and NPC1^I1061T^ homozygous fibroblasts 83.16 and 59413 were obtained from Dr. John O'Brien (Mayo Clinic, Rochester, MN). Normal skin fibroblasts AG10803, GM09503, GM05659 and GM03726 were obtained from the Coriell Cell Repository. All fibroblasts were cultured in DMEM containing 10% fetal bovine serum. Cells were harvested at 50–70% confluency.

### Microarray procedures and Data analysis

mRNA was isolated with the FastTrack 2.0 mRNA isolation kit according to the manufacturer's instructions (Invitrogen, Carlsbad, CA). A reference RNA comprised of 10 cell lines was used as the control for each hybridized sample (Stratagene, La Jolla, CA). Both sample and reference RNAs were amplified using the MessageAmp II aRNA amplification kit (Ambion, Austin, TX). Human cDNA microarrays consisting of ∼43,000 elements were obtained from the Stanford University functional genomics facility [58]. Hybridizations were conducted essentially as described [59]. The selected genes had >2 fold intensity ratio/background and were measurable in at least 80% of the data set. The resulting output was grouped by hierarchical clustering [60]. The two sets of genes were analyzed by significance analysis of microarrays (SAM) using 100 iterations in the two-class, unpaired data option [12].

### Immunoblots

Normal (AG10803) or NPC (GM03123) skin fibroblasts were washed 3 times with PBS, followed by incubation with RIPA buffer for 15 min. Lysates were centrifuged at 100,000 x g for 10 min and equal amounts of protein were analysed by immunoblot using commercially available antibodies. Signals were detected by chemiluminescence (Perkin Elmer Life Sciences Inc., Boston, MA). Antibodies and their sources were: Rabbit anti-FE65L1 (APPB2), Dr. Suzanne Guenette, Harvard Medical School; rabbit anti-BACE, Santa Cruz Biotechnology, Inc; Mouse anti-Nicastrin, BD transduction Laboratories; Rabbit anti-PSEN1 and APP-CT, Dr. William Mobley, Stanford School of Medicine.

### Data deposition

Original microarray data have been deposited in the Stanford Microarray Database: http://smd.stanford.edu

## Supporting Information

Table S1 Gene encoding channels, transporters and solute carriers that are upregulated in NPC fibroblasts.
(0.07 MB DOC)

Table S2 Genes involved in metal transport and homeostasis that are upregulated in NPC fibroblasts.
(0.03 MB DOC)

Table S3 Genes involved in membrane traffic and the cytoskeleton that are upregulated in NPC fibroblasts.
(0.09 MB DOC)

Dataset S1 This excel file presents SAM results for this data set, sorted alphabetically. False discovery rates are all <2%.
(2.47 MB XLS)
