# Supplementary material for: Clues to Neuro-Degeneration in Niemann-Pick Type C Disease from Global Gene Expression Profiling
Source: PLoS One. 2006 Dec 20;1(1):e19. doi: 10.1371/journal.pone.0000019 (PMC1762405; doi:10.1371/journal.pone.0000019)
Supplement: Table S1 — Gene encoding channels, transporters and solute carriers that are upregulated in NPC fibroblasts. (0.07 MB DOC) [file pone.0000019.s001.doc]

**Table S1.** Genes encoding channels, transporters and solute carriers that are upregulated in NPC fibroblasts.

| **Access. No.** | **Symbol** | **Name** | **Fold change** | **False Discovery Rate** |
| --- | --- | --- | --- | --- |
| AA486112 | ATP6V0E | ATPase, H+ transporting, lysosomal 9kDa, V0 subunit e | 1.5 | 1.9 |
| N33243 | ATP6V1C1 | ATPase, H+ transporting, lysosomal 42kDa, V1 subunit C, isoform 1 | 1.5 | 0.2 |
| AI053487 | CACNA1A | Calcium channel, voltage-dependent, P/Q type, alpha 1A subunit | 1.4 | 2.4 |
| AI016238 | CACNA1C | Calcium channel, voltage-dependent, L type, alpha 1C subunit | 1.5 | 0.1 |
| W73406 | CACNG1 | Calcium channel, voltage-dependent, gamma subunit 1 | 1.4 | 1.1 |
| AI146595 | CACNG4 | Calcium channel, voltage-dependent, gamma subunit 4 | 1.5 | 2 |
| AI675394 | CLCA2 | Chloride channel, calcium activated, family member 2 | 2.6 | 0 |
| AA488792 | CLCN6 | Chloride channel 6 | 1.5 | 0.2 |
| AA399181 | CLCNKA | Chloride channel Ka | 2 | 0.2 |
| AA125748 | KCMF1 | Potassium channel modulatory factor 1 | 1.4 | 0.9 |
| AA975384 | KCNA5 | Potassium voltage-gated channel, shaker-related subfamily, member 5 | 1.4 | 0.9 |
| AA776243 | KCNAB1 | Potassium voltage-gated channel, shaker-related subfamily, beta member 1 | 1.6 | 0.1 |
| AI654215 | KCNAB2 | Potassium voltage-gated channel, shaker-related subfamily, beta member 2 | 1.9 | 0.1 |
| AA069746 | KCNB1 | Potassium voltage-gated channel, Shab-related subfamily, member 1 | 1.6 | 0.4 |
| AA954569 | KCNC2 | Potassium voltage-gated channel, Shaw-related subfamily, member 2 | 1.6 | 1.5 |
| R15779 | KCND2 | Potassium voltage-gated channel, Shal-related subfamily, member 2 | 1.4 | 0.8 |
| AI017405 | KCNE3 | Potassium voltage-gated channel, Isk-related family, member 3 | 1.4 | 0.4 |
| AI160757 | KCNJ10 | Potassium inwardly-rectifying channel, subfamily J, member 10 | 2 | 0.1 |
| H16815 | KCNMB4 | Potassium large conductance Ca-activated channel, subfamily M, beta4 | 1.4 | 1.2 |
| AA443903 | KCNN4 | Potassium int./small conductance Ca-activated channel, subfamily N, member 4 | 1.5 | 0.3 |
| AI093325 | KCNQ3 | Potassium voltage-gated channel, KQT-like subfamily, member 3 | 2.1 | 0 |
| AI005321 | KCNU1 | Potassium channel, subfamily U, member 1 | 1.4 | 0.8 |
| AA455041 | KCTD7 | Potassium channel tetramerisation domain containing 7 | 1.6 | 0.2 |
| AA630035 | SCN9A | Sodium channel, voltage-gated, type IX, alpha | 2 | 0 |
| AI783618 | SLC10A2 | Solute carrier family 10 (sodium/bile acid cotransporter family), member 2 | 1.8 | 0.1 |
| AI261726 | SLC12A1 | Solute carrier family 12 (Na/potassium/chloride transporters), member 1 | 2 | 0.2 |
| T71008 | SLC13A5 | Solute carrier family 13 (sodium-dependent citrate transporter), member 5 | 1.4 | 2 |
| AA425395 | SLC16A2 | Solute carrier family 16 (monocarboxylic acid transporters), member 2 | 1.7 | 0.1 |
| AA858296 | SLC17A3 | Solute carrier family 17 (sodium phosphate), member 3 | 1.4 | 0.8 |
| AI056429 | SLC1A3 | DKFZP547J0410 protein | 1.7 | 0.2 |
| AI017670 | SLC22A6 | Solute carrier family 22 (organic anion transporter), member 6 | 1.8 | 0.1 |
| AA962217 | SLC23A3 | Solute carrier family 23 (nucleobase transporters), member 3 | 1.4 | 1.9 |
| AA052895 | SLC25A13 | Solute carrier family 25, member 13 (citrin) | 1.6 | 0.2 |
| AI344386 | SLC28A1 | Solute carrier family 28 (Na-coupled nucleoside transporter), member 1 | 1.7 | 0.4 |
| H02328 | SLC2A1 | Solute carrier family 2 (facilitated glucose transporter), member 1 | 1.4 | 1.7 |
| AA459296 | SLC34A2 | Solute carrier family 34 (sodium phosphate), member 2 | 2.2 | 0 |
| AA034501 | SLC35A3 | Solute carrier family 35 (UDP-N-acetylglucosamine (UDP-GlcNAc) transporter), member A3 | 1.4 | 2.8 |
| AA541579 | SLC35E1 | Solute carrier family 35, member E1 | 1.6 | 0.2 |
| R27212 | SLC41A2 | Solute carrier family 41, member 2 | 1.5 | 0.7 |
| R31315 | SLC43A2 | Solute carrier family 43, member 2 | 1.6 | 0.2 |
| AI306150 | SLC5A10 | Solute carrier family 5 (sodium/glucose cotransporter), member 10 | 1.6 | 0.2 |
| AA872711 | SLC5A12 | Solute carrier family 5 (sodium/glucose cotransporter), member 12 | 1.4 | 0.5 |
| AI688443 | SLC6A6 | Solute carrier family 6 (neurotransmitter transporter, taurine), member 6 | 1.4 | 0.8 |
| AA419176 | SLC7A5 | Solute carrier family 7 (cationic amino acid transporter, y+ system), mem. 5 | 1.8 | 0.8 |
| AA659627 | SLC7A8 | Solute carrier family 7 (cationic amino acid transporter, y+ system), mem. 8 | 1.4 | 1.9 |
| W47115 | SLC9A7 | Solute carrier family 9 (sodium/hydrogen exchanger), isoform 7 | 1.6 | 0.8 |
| N62948 | SLCO1A2 | Solute carrier organic anion transporter family, member 1A2 | 1.5 | 0.2 |
| AA534529 | SLCO4A1 | Solute carrier organic anion transporter family, member 4A1 | 2 | 0.2 |
