# Supplementary material for: Clues to Neuro-Degeneration in Niemann-Pick Type C Disease from Global Gene Expression Profiling
Source: PLoS One. 2006 Dec 20;1(1):e19. doi: 10.1371/journal.pone.0000019 (PMC1762405; doi:10.1371/journal.pone.0000019)
Supplement: Table S2 — Genes involved in metal transport and homeostasis that are upregulated in NPC fibroblasts. (0.03 MB DOC) [file pone.0000019.s002.doc]

**Table S2.** Genes involved in metal transport and homeostasis that are upregulated in NPC fibroblasts.

| **Access. No.** | **Symbol** | **Name** | **Fold change** | **False Discovery Rate** |
| --- | --- | --- | --- | --- |
| **Copper** |  |  |  |  |
| AA236141 | ATP7A | ATPase, Cu++ transporting, alpha polypeptide (Menkes syndrome) | 1.5 | 0.5 |
| N26536 | ATP7B | ATPase, Cu++ transporting, beta polypeptide (Wilson disease) | 1.5 | 0.8 |
| **Iron** |  |  |  |  |
| N71795 | FTH1 | Ferritin, heavy polypeptide 1 | 1.4 | 1.6 |
| AA971188 | FTL | Ferritin, light polypeptide | 1.7 | 0.7 |
| AA922939 | SFXN1 | Sideroflexin 1 | 1.8 | 0 |
| **Zinc** |  |  |  |  |
| AA149203 | SLC39A2 | Solute carrier family 39 (zinc transporter), member 2 | 1.9 | 0 |
