# Supplementary material for: Clues to Neuro-Degeneration in Niemann-Pick Type C Disease from Global Gene Expression Profiling
Source: PLoS One. 2006 Dec 20;1(1):e19. doi: 10.1371/journal.pone.0000019 (PMC1762405; doi:10.1371/journal.pone.0000019)
Supplement: Table S3 — Genes involved in membrane traffic and the cytoskeleton that are upregulated in NPC fibroblasts. (0.09 MB DOC) [file pone.0000019.s003.doc]

**Table S3.** Genes involved in membrane traffic and the cytoskeleton that are upregulated in NPC fibroblasts.

| **Access. No.** | **Symbol** | **Name** | **Fold change** | **False Discovery Rate** |
| --- | --- | --- | --- | --- |
| AA782337 | ANK2 | Ankyrin 2, neuronal | 1.5 | 0.4 |
| AA969184 | ANKRD1 | Ankyrin repeat domain 1 (cardiac muscle) | 1.9 | 1.7 |
| AI023136 | ANKRD26 | Ankyrin repeat domain 26 | 1.7 | 0.1 |
| R59488 | ANKRD6 | Ankyrin repeat domain 6 | 1.5 | 0.5 |
| AI049718 | ANKS1 | Ankyrin repeat and sterile alpha motif domain containing 1 | 1.4 | 1.1 |
| AI984289 | ANXA13 | Annexin A13 | 1.4 | 0.8 |
| AA872001 | ANXA6 | Annexin A6 | 1.6 | 0.8 |
| N78621 | AP1G1 | Adaptor-related protein complex 1, gamma 1 subunit | 1.4 | 2 |
| AA581123 | ARFGEF2 | ADP-ribosylation factor GEF2 (brefeldin A-inhibited) | 1.5 | 0.9 |
| AA878224 | ARHGAP26 | Rho GTPase activating protein 26 | 1.5 | 0.2 |
| AA905638 | ARHGAP28 | Rho GTPase activating protein 28 | 1.4 | 1.9 |
| AI342037 | ARHGAP8 | Rho GTPase activating protein 8 | 1.5 | 0.4 |
| H94857 | BLOC1S1 | Biogenesis of lysosome-related organelles complex-1, subunit 1 | 2.4 | 0 |
| AI050081 | CENTG2 | Trinucleotide repeat containing 17 | 1.6 | 0 |
| W37752 | CLN3 | Ceroid-lipofuscinosis, neuronal 3, juvenile (Batten, Spielmeyer-Vogt disease) | 1.4 | 1.6 |
| N73307 | CLN5 | Ceroid-lipofuscinosis, neuronal 5 | 1.4 | 0.5 |
| AA504455 | COG2 | Component of oligomeric golgi complex 2 | 1.6 | 0.4 |
| AA501982 | COG3 | Component of oligomeric golgi complex 3 | 1.5 | 0.8 |
| AA865234 | COPE | Coatomer protein complex, subunit epsilon | 2 | 0 |
| W47179 | CTSB | Cathepsin B | 1.5 | 1.3 |
| AA487231 | CTSH | Cathepsin H | 1.5 | 2.4 |
| AA873619 | DNAH5 | Dynein, axonemal, heavy polypeptide 5 | 1.9 | 0.2 |
| AA448927 | DNAJC1 | DnaJ (Hsp40) homolog, subfamily C, member 1 | 1.5 | 0.7 |
| N38963 | DNCI2 | Dynein, cytoplasmic, intermediate polypeptide 2 | 1.7 | 0.5 |
| AA974905 | FSCN3 | Fascin homolog 3, actin-bundling protein, testicular | 1.9 | 0 |
| AA159770 | KIF1C | Kinesin family member 1C | 1.4 | 0.8 |
| H28973 | LAMP1 | Lysosomal-associated membrane protein 1 | 1.4 | 1.7 |
| AA625666 | LITAF | Lipopolysaccharide-induced TNF factor | 1.4 | 1.3 |
| AI261935 | LRP6 | Low density lipoprotein receptor-related protein 6 | 2.7 | 0 |
| AI290482 | M6PR | Mannose-6-phosphate receptor (cation dependent) | 1.5 | 0.4 |
| AA570622 | MICAL-L2 | MICAL-like 2 | 2.4 | 0.2 |
| AI371190 | MYBPC3 | Myosin binding protein C, cardiac | 1.5 | 0.4 |
| AA461174 | MYBPH | Myosin binding protein H | 1.5 | 0.2 |
| H79798 | MYBPHL | Myosin binding protein H-like | 1.5 | 0.2 |
| AA662473 | MYH11 | Myosin, heavy polypeptide 11, smooth muscle | 2.1 | 0 |
| AA488346 | MYL6 | Myosin, light polypeptide 6, alkali, smooth muscle/non-muscle | 1.4 | 1.6 |
| AI817883 | MYO1A | Myosin IA | 1.7 | 0.2 |
| AI360772 | MYO1F | Myosin IF | 1.5 | 0.2 |
| AI186799 | MYOM2 | Myomesin (M-protein) 2, 165kDa | 1.4 | 0.8 |
| AI301081 | NDFIP2 | Nedd4 family interacting protein 2 | 2 | 0 |
| AA442095 | NEDD4 | Neural precursor cell expressed, developmentally down-regulated 4 | 1.5 | 0.5 |
| AA458578 | NEDD4L | Neural precursor cell expressed, developmentally down-regulated 4-like | 1.4 | 1.6 |
| AA740404 | NSF | N-ethylmaleimide-sensitive factor | 2.6 | 0 |
| AA600325 | RAB10 | RAB10, member RAS oncogene family | 1.6 | 0.2 |
| AI299673 | RAB20 | RAB20, member RAS oncogene family | 1.7 | 0.2 |
| AA598440 | RAB27A | RAB27A, member RAS oncogene family | 1.3 | 2 |
| H69004 | RAB36 | RAB36, member RAS oncogene family | 1.4 | 1.2 |
| R05931 | RAB37 | RAB37, member RAS oncogene family | 1.5 | 0.8 |
| AA705288 | RAB39B | RAB39B, member RAS oncogene family | 1.5 | 0.4 |
| AA995055 | RAB7B | RAB7B, member RAS oncogene family | 1.4 | 0.8 |
| AI375563 | RAB9P40 | Rab9 effector p40 | 1.3 | 3.2 |
| AA283925 | RABGEF1 | RAB guanine nucleotide exchange factor (GEF) 1 | 1.5 | 0.3 |
| AI015265 | RABL2B | RAB, member of RAS oncogene family-like 2B | 1.4 | 0.2 |
| R32801 | SCAMP2 | Secretory carrier membrane protein 2 | 2 | 0.1 |
| AW075462 | SCAMP3 | Secretory carrier membrane protein 3 | 1.6 | 0.2 |
| AI262350 | SCAMP4 | Hypothetical protein BC011824 | 1.5 | 0.9 |
| W47156 | SEC22L2 | SEC22 vesicle trafficking protein-like 2 (S. cerevisiae) | 1.5 | 0.9 |
| H64779 | SEC24A | SEC24 related gene family, member A (S. cerevisiae) | 1.4 | 1.3 |
| T40211 | SEC31L1 | SEC31-like 1 (S. cerevisiae) | 1.6 | 0.2 |
| AA953674 | SEC3L1 | SEC3-like 1 (S. cerevisiae) | 1.7 | 0.4 |
| AA621236 | SEC61A2 | Sec61 alpha 2 subunit (S. cerevisiae) | 1.4 | 1.7 |
| W96106 | SEC61G | Sec61 gamma subunit | 1.6 | 2 |
| AI265975 | SORCS1 | Sortilin-related VPS10 domain containing receptor 1 | 2.1 | 0 |
| AA974248 | SORCS2 | Sortilin-related VPS10 domain containing receptor 2 | 1.5 | 0.3 |
| AI082431 | TBC1D16 | TBC1 domain family, member 16 | 1.9 | 0.1 |
| N58164 | TBC1D17 | TBC1 domain family, member 17 | 1.6 | 0.4 |
| AI005114 | TBC1D22A | TBC1 domain family, member 22A | 1.6 | 0.4 |
| H49972 | VAMP2 | Vesicle-associated membrane protein 2 (synaptobrevin 2) | 1.7 | 0.1 |
| N66593 | VMP | Vesicular membrane protein p24 | 1.5 | 0.9 |
| N53520 | VPS13C | Vacuolar protein sorting 13C (yeast) | 1.5 | 0.4 |
| AA699313 | VTI1A | Vesicle transport through interaction with t-SNAREs homolog 1A | 1.5 | 0.2 |
